# Recommendations for standardizing nomenclature for dietary (poly)phenol catabolites

**DOI:** 10.1093/ajcn/nqaa204

**Published:** 2020-09-16

**Authors:** Colin D Kay, Michael N Clifford, Pedro Mena, Gordon J McDougall, Cristina Andres-Lacueva, Aedin Cassidy, Daniele Del Rio, Nikolai Kuhnert, Claudine Manach, Gema Pereira-Caro, Ana Rodriguez-Mateos, Augustin Scalbert, Francisco Tomás-Barberán, Gary Williamson, David S Wishart, Alan Crozier

**Affiliations:** Plants for Human Health Institute, Food Bioprocessing and Nutrition Sciences Department, North Carolina State University, Kannapolis, NC, USA; School of Bioscience and Medicine, University of Surrey, Guildford, United Kingdom; Department of Nutrition, Dietetics and Food, Monash University, Notting Hill, Victoria, Australia; Department of Food and Drugs, University of Parma, Parma, Italy; The James Hutton Research Institute, Invergowrie, Dundee, United Kingdom; Department of Nutrition, Food Science and Gastronomy, University of Barcelona, Barcelona, Spain; CIBER Frailty and Healthy Aging (CIBERfes), Institute of Health Carlos III, Barcelona, Spain; Institue for Food Security, Queen's University, Belfast, United Kingdom; Department of Food and Drugs, University of Parma, Parma, Italy; Department of Life Sciences and Health, Jacobs University, Bremen, Germany; Université Clermont Auvergne, INRAE, Clermont-Ferrand, France; Department of Food Science and Health, Andalusian Institute of Agricultural and Fisheries Research and Training, Cordoba, Spain; Department of Nutritional Sciences, Kings College, London, United Kingdom; International Agency for Research on Cancer, WHO, Lyon, France; Food and Health Laboratory, CEBAS-CSIC, Espinardo University Campus, Murcia, Spain; Department of Nutrition, Dietetics and Food, Monash University, Notting Hill, Victoria, Australia; Department of Biological Sciences, University of Alberta, Edmonton, Canada; School of Medicine, Dentistry and Nursing, University of Glasgow, Glasgow, United Kingdom; Department of Nutrition, University of California, Davis, Davis, CA, USA

**Keywords:** dietary (poly)phenols, microbial catabolites, phase II metabolites, nomenclature, food/diet metabolome

## Abstract

There is a lack of focus on the protective health effects of phytochemicals in dietary guidelines. Although a number of chemical libraries and databases contain dietary phytochemicals belonging to the plant metabolome, they are not entirely relevant to human health because many constituents are extensively metabolized within the body following ingestion. This is especially apparent for the highly abundant dietary (poly)phenols, for which the situation is compounded by confusion regarding their bioavailability and metabolism, partially because of the variety of nomenclatures and trivial names used to describe compounds arising from microbial catabolism in the gastrointestinal tract. This confusion, which is perpetuated in online chemical/metabolite databases, will hinder future discovery of bioactivities and affect the establishment of future dietary guidelines if steps are not taken to overcome these issues. In order to resolve this situation, a nomenclature system for phenolic catabolites and their human phase II metabolites is proposed in this article and the basis of its format outlined. Previous names used in the literature are cited along with the recommended nomenclature, International Union of Pure and Applied Chemistry terminology, and, where appropriate, Chemical Abstracts Service numbers, InChIKey, and accurate mass.

## Introduction

The complexity of foods and the accompanying limited information on dietary phytochemicals in metabolomics and dietary guidelines has recently been recognized (1). Although a number of databases contain information on phytochemicals of the plant metabolome, they are not directly relevant to human health or metabolomics because many of these compounds undergo substantial metabolism within the body following ingestion. Chemical/metabolite databases such as Phenol-Explorer, FooDB, ChEBI, and PubChem, metabolomics data repositories including HMDB and MetaboLights, and mass spectra databases such as Mass Bank, ReSpect for Phytochemicals, Golm, and Metlin contain fragmentary information that is far from a comprehensive overview of human metabolites resulting from consumption of phytochemicals. Plant-derived dietary compounds have a much greater metabolomic complexity in vivo than is currently recognized and, as a consequence, the metabolomes derived from plant foods are undercharacterized. This is evident with the highly abundant dietary (poly)phenols, which are among the phytochemicals that are recognized as important regulators of human health (2, 3). To further complicate matters, there are areas of confusion regarding the absorption and metabolism of dietary (poly)phenols, partially because of the variety of nomenclatures and trivial names used to describe compounds that arise in the gastrointestinal tract as the result of microbial catabolism and human phase II metabolism. This confusion is perpetuated in online chemical and metabolomic databases.

One of the current goals of (poly)phenol research is to establish dietary guidance including a recommended intake or number of servings of (poly)phenol-rich foods to promote optimal health or reduce disease risk (4, 5). The aim of the proposed nomenclature standardization is to advance basic and applied (poly)phenol research, and associated methods such as metabolome pathway analysis tools (6–8), to provide evidence in support of observational studies (epidemiological evidence) that form the foundation for Dietary Guidelines (National Nutrition Monitoring and Related Research Act). Databases and pathway tools/systems operate using compound recognition, which requires standardized nomenclature and machine-readable identifiers such as InChIKey, and Chemical Abstracts Service (CAS) numbers. It is therefore paramount for (poly)phenol researchers to standardize nomenclature and reporting practices. In addition, there is a requirement for a reliable system of translation, permitting incorporation of pre-existing data described using older or nonstandard terminology into modern databases and bioinformatics tools such as PubChem and HMDB. This need to adopt universally accepted annotations, classification schemes and ontologies, facilitating data interoperability in metabolomics, has recently been emphasized (9, 10).

Calls for standardizing reporting practices in (poly)phenol research are not novel. In 2015 a special article was published in *The American Journal of Clinical Nutrition*, in which recommendations were proposed on reporting requirements for flavonoids in research (11). This report focused primarily on standardization of reporting relevant to dietary assessment, food composition characterization, food administration, human and animal intervention study design, and analytical reporting. Subsequently, a joint International Union of Pure and Applied Chemistry (IUPAC)/International Union of Biochemistry and Molecular Biology working group published recommendations for general nomenclature of (poly)phenols and provided examples of acceptable trivial names, together with semisystematic and fully systematic names (12). However, both these publications focused on phytochemical nomenclature used to characterize secondary plant metabolites in foods and did not cover catabolites arising from the gut microbiome. The current special article provides standardized nomenclature and reporting practices for human metabolites of dietary (poly)phenols encompassing gut microbial catabolites and their human phase II metabolites, which were overlooked in the previous reports. Our aim is to eliminate inconsistencies in nomenclature/terminology, which create confusion and ultimately affects interpretation of data surrounding the biological effects of plant-based foods in epidemiology and nutrition intervention studies.

## Standardization

A call for standardized nomenclature and reporting practices is timely because it will undoubtedly help researchers establish the untapped potential of the non-nutrient phytochemicals in food and diet to modulate human health. Phytochemical structural data need to be captured in ways that have future utility, and this will become of increasing importance because national funding bodies are beginning to require studies make their source data publicly available using open access data repositories (8, 13). Progress is currently impeded by confusion in the nutrition and food science literature because of the previously mentioned inconsistent chemical nomenclatures applied to phenolic catabolites, which make it very difficult to integrate data for review and meta-analysis. The magnitude of this problem is clearly illustrated by the results of Web of Science Core database searches (February 13, 2020; https://clarivate.com/webofsciencegroup/solutions/web-of-science) using known variations in nomenclature for a common (poly)phenol catabolite as the search “topic.” This revealed that even differences in hyphenation have significant impact on how many references may be identified when searching for a single compound. A search using our recommended name “3-(3′-hydroxyphenyl)propanoic acid” (**Table 1**) yielded fifty four references, but its associated synonyms yielded additional references as follows: 3-hydroxyphenyl-propionic acid (thirty references), 3-hydroxy-phenylpropionic acid (one reference), 3-hydroxy-phenyl-propionic acid (two references), and 3-(3′-hydroxy-phenyl)-propionic acid (three references). Similarly, a further search using the recommended name “3-hydroxy-3-(3′-hydroxyphenyl)propanoic acid” (Table 1) produced only one reference, whereas its synonyms 3-hydroxy-(3′-hydroxyphenyl)propionic acid yielded eight references, 3-hydroxyphenyl-hydracrylic acid three references, and 3-hydroxyphenylhydracrylic acid one reference. The nomenclature system for phenolic catabolites provided herein will help clarify confusion in the nutrition literature and provide a harmonized approach for online databases.

**TABLE 1:** Examples of recommended nomenclature for (poly)phenols, their microbial catabolites, and human phase II conjugates^1^

Recommended names^2^	Synonyms and incorrect nomenclature^2,3^	CAS no.^4^	IUPAC^5^ (InChIKey;^6^ monoisotopic mass g/mol)
1. Benzene diols and triols (C_6_–C_0_)			
Benzene-1,2-diol	1,2-Dihydroxybenzene	120-80-9	Benzene-1,2-diol
	Catechol		(YCIMNLLNPGFGHC-UHFFFAOYSA-N;
	Pyrocatechol		110.036779 g/mol)
	Brenzcatechin		
Benzene-1,3-diol	1,3-DihydroxybenzeneResorcinol	108-46-3	Benzene-1,3-diol(GHMLBKRAJCXXBS-UHFFFAOYSA-N; 110.036779 g/mol)
Benzene-1,2,3-triol	1,2,3-Trihydroxybenzene	87-66-1	Benzene-1,2,3-triol
	Pyrogallol		(WQGWDDDVZFFDIG-UHFFFAOYSA-N; 126.031694 g/mol)
Benzene-1,3,5-triol	1,3,5-Trihydroxybenzene	108-73-6	Benzene-1,3,5-triol
	Phloroglucinol		(QCDYQQDYXPDABM-UHFFFAOYSA-N; 126.031694 g/mol)
2. Benzaldehydes (C_6_–C_1_)			
4-Hydroxybenzaldehyde	4-Formylphenol	123-08-0	4-Hydroxybenzaldehyde (RGHHSNMVTDWUBI-UHFFFAOYSA-N; 122.036779 g/mol)
3,4-Dihydroxybenzaldehyde	Protocatechualdehyde	139-85-5	3,4-Dihydroxybenzaldehyde (IBGBGRVKPALMCQ-UHFFFAOYSA-N; 138.031694 g/mol)
2,4,6-Trihydroxybenzaldehyde	Phloroglucinaldehyde	487-70-7	2,4,6-Trihydroxybenzaldehyde (BTQAJGSMXCDDAJ-UHFFFAOYSA-N; 154.026609 g/mol)
3-Hydroxy-4-methoxybenzaldehyde	Isovanillin	621-59-0	3-Hydroxy-4-methoxybenzaldehyde (JVTZFYYHCGSXJV-UHFFFAOYSA-N; 152.047344 g/mol)
4-Hydroxy-3-methoxybenzaldehyde	3-Methoxy-4-hydroxybenzaldehyde	121-33-5	4-Hydroxy-3-methoxybenzaldehyde
	Vanillin		(MWOOGOJBHIARFG-UHFFFAOYSA-N; 152.047344 g/mol)
3. Benzoic acids (C_6_–C_1_)			
3-Hydroxybenzoic acid	3-Hydroxybenzene carboxylic acid	99-06-9	3-Hydroxybenzoic acid (IJFXRHURBJZNAO-UHFFFAOYSA-N; 138.031694 g/mol)
4-Hydroxybenzoic acid	4-Hydroxybenzene carboxylic acid	99-96-7	4-Hydroxybenzoic acid (FJKROLUGYXJWQN-UHFFFAOYSA-N; 138.031694 g/mol)
2,5-Dihydroxybenzoic acid	Gentisic acid	490-79-9	2,5-Dihydroxybenzoic acid
	5-Hydroxy-salicylic acid		(WXTMDXOMEHJXQO-UHFFFAOYSA-N;
	Hydroquinone carboxylic acid		154.026611 g/mol)
3,4-Dihydroxybenzoic acid	Protocatechuic acid	99-50-3	3,4-Dihydroxybenzoic acid (YQUVCSBJEUQKSH-UHFFFAOYSA-N; 154.026609 g/mol)
3,5-Dihydroxybenzoic acid	α-Resorcylic acid	99-10-5	3,5-Dihydroxybenzoic acid (UYEMGAFJOZZIFP-UHFFFAOYSA-N; 154.026609 g/mol)
3,4,5-Trihydroxybenzoic acid	Gallic acid	149-91-7	3,4,5-Trihydroxybenzoic acid (LNTHITQWFMADLM-UHFFFAOYSA-N; 170.021523 g/mol)
2-Amino-5-hydroxybenzoic acid	5-Hydroxyanthranilic acid	394-31-0	2-Amino-5-hydroxybenzoic acid (HYNQTSZBTIOFKH-UHFFFAOYSA-N; 153.042593 g/mol)
Benzoic acid-4-glucuronide	—	—	—
Benzoic acid-4-sulfate	—	—	—
3-Hydroxybenzoic acid-4-glucuronide	[*Protocatechuic acid-4-glucuronide*]	—	—
4-Hydroxybenzoic acid-3-glucuronide	[*Protocatechuic acid-3-glucuronide*]	—	—
3-Hydroxybenzoic acid-4-sulfate	[*Protocatechuic acid-4-sulfate*]	—	—
4-Hydroxybenzoic acid-3-sulfate	[*Protocatechuic acid-3-sulfate*]	—	—
3-Hydroxy-4-methoxybenzoic acid	Isovanillic acid	645-08-9	3-Hydroxy-4-methoxybenzoic acid (LBKFGYZQBSGRHY-UHFFFAOYSA-N; 168.042259 g/mol)
4-Methoxybenzoic acid-3-glucuronide	[*Isovanillic acid-3-glucuronide*]	—	—
4-Methoxybenzoic acid-3-sulfate	[*Isovanillic acid-3-sulfate*]	—	—
4-Hydroxy-3-methoxybenzoic acid	Vanillic acid	499-76-3	4-Hydroxy-3-methylbenzoic acid (LTFHNKUKQYVHDX-UHFFFAOYSA-N; 152.047344 g/mol)
3-Methoxybenzoic acid-4-glucuronide	[*Vanillic acid-4-glucuronide*]	—	—
3-Methoxybenzoic acid-4-sulfate	[*Vanillic acid-4-sulfate*]	—	—
3,5-Dimethoxy-4-hydroxybenzoic acid	Syringic acid	530-57-4	4-Hydroxy-3,5-dimethoxybenzoic acid (JMSVCTWVEWCHDZ-UHFFFAOYSA-N;198.052823 g/mol)
3,4-Dimethoxybenzoic acid	Veratric acid	93-07-2	3,4-Dimethoxybenzoic acid (DAUAQNGYDSHRET-UHFFFAOYSA-N;182.057909 g/mol)
4. Cinnamic acids (C_6_–C_3_ unsaturated)^7^			
Cinnamic acid	*trans*-Cinnamic acid	621-82-9, 140-10-3	(2*E*)-3-phenylprop-2-enoic acid (WBYWAXJHAXSJNI-VOTSOKGWSA-N;148.052429 g/mol)
2′-Hydroxycinnamic acid	2-Hydroxycinnamic acid^8^	614-60-8	(2*E*)-3-(2-hydroxyphenyl)prop-2-enoic acid
	*o*-Coumaric acid		(PMOWTIHVNWZYFI-AATRIKPKSA-N;
	*tran*s-2-Coumaric acid		164.047344 g/mol)
	*tran*s-2-Hydroxycinnamic acid		
	3-(2-Hydroxyphenyl)acrylic acid		
3′-Hydroxycinnamic acid	3-Hydroxycinnamic acid^8^	588-30-7	(2*E*)-3-(3-hydroxyphenyl)prop-2-enoic acid
	*m*-Coumaric acid		(KKSDGJDHHZEWEP-SNAWJCMRSA-N;
	3-Coumaric acid		164.047344 g/mol)
	*trans*-3-Coumaric acid		
	3-(3-Hydroxyphenyl)acrylic acid		
4′-Hydroxycinnamic acid	4-Hydroxycinnamic acid^8^	501-98-4	(2*E*)-3-(4-hydroxyphenyl)prop-2-enoic acid
	Coumaric acid		(NGSWKAQJJWESNS-ZZXKWVIFSA-N;
	*p*-Coumaric acid		164.047344 g/mol)
	4-Coumaric acid		
	*trans*-4-Hydroxycinnamic acid		
	(4-Hydroxyphenyl)acrylic acid		
3′,4′-Dihydroxycinnamic acid	3,4-Dihydroxycinnamic acid^8^	331-39-5,	(2*E*)-3-(3,4-dihydroxyphenyl)prop-2-enoic acid
	Caffeic acid	501-16-6	(QAIPRVGONGVQAS-DUXPYHPUSA-N;
	*trans*-Caffeic acid		180.042259 g/mol)
	3-(3,4-Dihydroxyphenyl)acrylic acid		
Cinnamic acid-4′*-*glucuronide	Cinnamic acid-4*-*glucuronide^8^		
	[p*-Coumaric acid-4′-glucuronide*]		
4′-Hydroxy-3′-methoxycinnamic acid	4-Hydroxy-3-methoxycinnamic acid^8^Ferulic acid	537-98-4	(2*E*)-3-(4-hydroxy-3-methoxyphenyl)prop-2-enoic acid (KSEBMYQBYZTDHS-HWKANZROSA-N;
	*trans*-Ferulic acid		194.057909 g/mol)
	3-(4-Hydroxy-3-methoxyphenyl)acrylic acid		
3′-Methoxycinnamic acid-4′-glucuronide	3-Methoxycinnamic acid-4-glucuronide^8^	—	—
	[*Ferulic acid-4-glucuronide*]		
3′-Methoxycinnamic acid-4′-sulfate	3-Methoxycinnamic acid-4-sulfate^8^	—	—
	[*Ferulic acid-4′-sulfate*]		
	[*Ferulic acid-4-sulfate*]		
3′-Hydroxy-4′-methoxycinnamic acid	3-Hydroxy-4-methoxycinnamic acid^8^Isoferulic acid	537-73-5	(2*E*)-3-(3-hydroxy-4-methoxyphenyl)prop-2-enoic acid(QURCVMIEKCOAJU-HWKANZROSA-N;
	Hesperetate		194.057909 g/mol)
	3-(3-Hydroxy-4-methoxyphenyl)acrylic acid		
4′-Methoxycinnamic acid-3′*-*glucuronide	4-Methoxycinnamic acid-3*-*glucuronide^8^	—	—
	[*Isoferulic acid-3′-glucuronide*]		
	[*Isoferulic acid-3-glucuronide*]		
4′-Methoxycinnamic acid-3′*-*sulfate	4-Methoxycinnamic acid-3*-*sulfate^8^	—	—
	[*Isoferulic acid-3′-sulfate*]		
	[*Isoferulic acid-3-sulfate*]		
5. Phenylpropanoic acids (C_6_–C_3_)			
3-Phenylpropanoic acid	3-(Phenyl)propionic acid[*Dihydrocinnamic acid*]	501-52-0	3-Phenylpropanoic acid (XMIIGOLPHOKFCH-UHFFFAOYSA-N;150.06808 g/mol)
3-(3′-Hydroxyphenyl)propanoic acid	3-(3-Hydroxyphenyl)propanoic acid^8^	621-54-5	3-(3-Hydroxyphenyl)propanoic acid
	3-(3-Hydroxyphenyl)propionic acid		(QVWAEZJXDYOKEH-UHFFFAOYSA-N;
	3-Hydroxy-dihydrocinnamic acid		166.062994 g/mol)
	*m*-Hydroxy-dihydrocinnamic acid		
3-(4′-Hydroxyphenyl)propanoic acid	3-(4-Hydroxyphenyl)propanoic acid^8^	10516-71-9	3-(4-Hydroxyphenyl)propanoic acid
	3-(4-Hydroxyphenyl)propionic acid		(NMHMNPHRMNGLLB-UHFFFAOYSA-N;
	4-Hydroxy-dihydrocinnamic acid		180.078644 g/mol)
	*m*-Hydroxy-dihydrocinnamic acid		
	Phloretic acid		
3-(3′,4′-Dihydroxyphenyl)propanoic acid	3-(3,4-Dihydroxyphenyl)propanoic acid^8^	1078-61-1	3-(3,4-Dihydroxyphenyl)propanoic acid
	3-(3,4-Dihydroxyphenyl)propionic acid		(DZAUWHJDUNRCTF-UHFFFAOYSA-N;
	Dihydrocaffeic acid		182.057909 g/mol)
	3,4-Dihydroxybenzenepropanoic acid		
	3,4-Dihydroxy-dihydrocinnamic acid		
3-(3′,5′-Dihydroxyphenyl)propanoic acid	3-(3,5-Dihydroxyphenyl)propanoic acid^8^	26539-01-5	3-(3,5-Dihydroxyphenyl)propanoic acid
	3-(3,5-Dihydroxyphenyl)propionic acid		(ITPFIKQWNDGDLG-UHFFFAOYSA-N;
	3,5-Dihydroxybenzenepropanoic acid		182.057909 g/mol)
	3,5-Dihydroxy-dihydrocinnamic acid		
3-(Phenyl)propanoic acid-4′-glucuronide	3-(Phenyl)propanoic acid-4-glucuronide^8^	—	—
	3-(Phenyl)propionic acid-4-glucuronide		
	[*4-Hydroxy-dihydrocinnamic acid-4-glucuronide*]		
	[p*-Hydroxy-dihydrocinnamic acid-glucuronide*]		
3-(3′-Methoxyphenyl)propanoic acid	3-(3-Methoxyphenyl)propanoic acid^8^	1798-09-0	3-(3-Methoxyphenyl)propanoic acid
	3-(3-Methoxyphenyl)propionic acid		(BJJQJLOZWBZEGA-UHFFFAOYSA-N;
	3-Methoxy-dihydrocinnamic acid		166.062994 g/mol)
3-(4′-Methoxyphenyl)propanoic acid	3-(4-Methoxyphenyl)propanoic acid^8^	1929-29-9	3-(4-Methoxyphenyl)propanoic acid
	3-(4-Methoxyphenyl)propionic acid		(FIUFLISGGHNPSM-UHFFFAOYSA-N;
	4-Methoxy-dihydrocinnamic acid		180.078644 g/mol)
3-(3′-Hydroxy-4′-methoxyphenyl)propanoic acid	3-(3-Hydroxy-4-methoxyphenyl)propanoic acid^8^	1135-15-5	3-(3-Hydroxy-4-methoxyphenyl)propanoic acid
	Dihydro-isoferulic acid		(ZVIJTQFTLXXGJA-UHFFFAOYSA-N;
	3-(3-Hydroxy-4-methoxyphenyl)dihydrocinnamic acid		196.073559 g/mol)
3-(4′-Hydroxy-3′-methoxyphenyl)propanoic acid	3-(4-Hydroxy-3-methoxyphenyl)propanoic acid^8^	1135-23-5	3-(4-Hydroxy-3-methoxyphenyl) propanoic acid
	3-(4-Hydroxy-3-methoxyphenyl)propionic acid		(BOLQJTPHPSDZHR-UHFFFAOYSA-N;
	Dihydroferulic acid		196.073559 g/mol)
	3-(4-Hydroxy-3-methoxyphenyl)dihydrocinnamic acid		
3-(3′-Hydroxyphenyl)propanoic acid-4′-glucuronide	3-(3-Hydroxyphenyl)propanoic acid-4-glucuronide^8^	—	—
	3-(3-Hydroxyphenyl)propionic acid-4-glucuronide		
	[*Dihydrocaffeic acid-4′-glucuronide*]		
	[*Dihydrocaffeic acid-4-glucuronide*]		
3-(3′-Hydroxyphenyl)propanoic acid-4′-sulfate	3-(3-Hydroxyphenyl)propanoic acid-4-sulfate^8^	—	—
	3-(3-Hydroxyphenyl)propionic acid-4-sulfate		
	[*Dihydrocaffeic acid-4′-sulfate*]		
	[*Dihydrocaffeic acid-4-sulfate*]		
3-(4′-Hydroxyphenyl)propanoic acid-3′-glucuronide	3-(4-Hydroxyphenyl)propionic acid-3-glucuronide^8^	—	—
	3-(4-Hydroxyphenyl)propanoic acid-3-glucuronide		
	[*Dihydrocaffeic acid-3′-glucuronide*]		
	[*Dihydrocaffeic acid-3-glucuronide*]		
3-(4′-Hydroxyphenyl)propanoic acid-3′-sulfate	3-(4-Hydroxyphenyl)propanoic acid-3-sulfate^8^	—	—
	3-(4-Hydroxyphenyl)propionic acid-3-sulfate		
	[*Dihydrocaffeic acid-3′-sulfate*]		
	[*Dihydrocaffeic acid-3-sulfate*]		
3-(3′-Methoxyphenyl)propanoic acid-4′-glucuronide	3-(3-Methoxyphenyl)propanoic acid-4-glucuronide^8^	—	—
	3-(3-Methoxyphenyl)propionic acid-4-glucuronide		
	[*Dihydroferulic acid-4′-glucuronide*]		
	[*Dihydroferulic acid-4-glucuronide*]		
3-(3′-Methoxyphenyl)propanoic acid-4′-sulfate	3-(3-Methoxyphenyl)propanoic acid-4-sulfate^8^	—	—
	3-(3-Methoxyphenyl)propionic acid-4-sulfate		
	[*Dihydroferulic acid-4′-sulfate*]		
	[*Dihydroferulic acid-4-sulfate*]		
3-(4′-Methoxyphenyl)propanoic acid-3′-glucuronide	3-(4-Methoxyphenyl)propanoic acid-3-glucuronide^8^	—	—
	3-(4-Methoxyphenyl)propionic acid-3-glucuronide		
	[*Dihydro-isoferulic acid-3′-glucuronide*]		
3-(4′-Methoxyphenyl)propanoic acid-3′-sulfate	3-(4-Methoxyphenyl)propanoic acid-3-sulfate^8^	—	—
	3-(4-Methoxyphenyl)propionic acid-3-sulfate		
	[*Dihydro-isoferulic acid-3′-sulfate*]		
	[*Dihydro-isoferulic acid-3-sulfate*]		
6. Hydroxy-3-(phenyl)propanoic acids (C_6_–C_3_)^9^			
3-Hydroxy-3-(phenyl)propanoic acid	3-(Phenyl)-3-hydroxypropionic acid	3480-87-3	3-Hydroxy-3-phenylpropanoic acid
	3-(Phenyl)hydracrylic acid		(AYOLELPCNDVZKZ-UHFFFAOYSA-N;166.062994 g/mol)
3-Hydroxy-3-(3′-hydroxyphenyl)propanoic acid	3-Hydroxy-3-(3-hydroxyphenyl)propanoic acid^8^	3247-75-4	3-Hydroxy-3-(3-hydroxyphenyl)propanoic acid
	3-(3-Hydroxyphenyl)-3-hydroxypropionic acid		(KHTAGVZHYUZYMF-UHFFFAOYSA-N;
	3-Hydroxy-3-(3′-hydroxyphenyl)propionic acid		182.057909 g/mol)
	3-Hydroxy-3-(3-hydroxyphenyl)propionic acid		
	β,*m*-Dihydroxyphenylpropionic acid		
	3-(3-Hydroxyphenyl)hydracrylic acid		
	3-(3′-Hydroxyphenyl)hydracrylic acid		
	*m*-Hydroxyphenylhydracrylic acid		
3-Hydroxy-3-(3′-hydroxy-4′-methoxyphenyl)propanoic acid	3-Hydroxy-3-(3-hydroxy-4-methoxyphenyl)propanoic acid^8^	—	3-Hydroxy-3-(3-hydroxy-4-methoxyphenyl)propanoic acid
	3-Hydroxy-3-(3-hydroxy-4-methoxyphenyl)propionic acid		(JEXBTMWMYGBBHO-UHFFFAOYSA-N;
	3-(3-Hydroxy-4-methoxyphenyl)-3-hydroxypropanoic acid		212.068473 g/mol)
	3-(3-Hydroxy-4-methoxyphenyl)-3-hydroxypropionic acid		
	3-(3′-Hydroxy-4′-methoxyphenyl)hydracrylic acid		
	3-(3-Hydroxy-4-methoxyphenyl)hydracrylic acid		
2-Hydroxy-3-(phenyl)propanoic acid	3-(Phenyl)-2-hydroxypropanoic acid^8^	828-01-3	2-Hydroxy-3-phenylpropionic acid
	2-Hydroxy-3-(phenyl)propionic acid		(VOXXWSYKYCBWHO-UHFFFAOYSA-N;
	3-(Phenyl)-2-hydroxypropionic acid		166.062994 g/mol)
	3-(Phenyl)lactic acid		
2-Hydroxy-3-(4′-hydroxyphenyl)propanoic acid	2-Hydroxy-3-(4-hydroxyphenyl)propanoic acid^8^	306-23-0	2-Hydroxy-3-(4-hydroxyphenyl)propanoic acid
	3-(4-Hydroxyphenyl)-2-hydroxypropanoic acid		(JVGVDSSUAVXRDY-UHFFFAOYSA-N;
	2-Hydroxy-3-(4-hydroxyphenyl)propionic acid		182.057909 g/mol)
	3-(4′-Hydroxyphenyl)-2-hydroxypropionic acid		
	3-(4′-Hydroxyphenyl)lactic acid		
	3-(4-Hydroxyphenyl)lactic acid		
2-Hydroxy-3-(3′,4′-dihydroxyphenyl)propanoic acid	2-Hydroxy-3-(3,4-dihydroxyphenyl)propanoic acid^8^	23028-17-3	3-(3,4-Dihydroxyphenyl)-2-hydroxypropanoic acid
	3-(3,4-Dihydroxyphenyl)-2-hydroxypropanoic acid		(PAFLSMZLRSPALU-UHFFFAOYSA-N;
	2-Hydroxy-3-(3′,4′-dihydroxyphenyl)propionic acid		198.052823 g/mol)
	3-(3,4-Dihydroxyphenyl)-2-hydroxypropionic acid		
	3-(3′,4′-Dihydroxyphenyl)lactic acid		
	3-(3,4-Dihydroxyphenyl)lactic acid		
7. Phenylacetic acids (C_6_–C_2_)			
Phenylacetic acid	Phenylethanoic acid	103-82-2	2-Phenylacetic acid
	2-Phenylethanoate		(WLJVXDMOQOGPHL-UHFFFAOYSA-N;136.052429 g/mol)
3′-Hydroxyphenylacetic acid	3-Hydroxyphenylacetic acid^8^	621-37-4	2-(3-Hydroxyphenyl)acetic acid
	3-Hydroxyphenylethanoic acid		(FVMDYYGIDFPZAX-UHFFFAOYSA-N;152.047344 g/mol)
3′-Methoxyphenylacetic acid	3-Methoxyphenylacetic acid^8^	1798-09-0	2-(3-Methoxyphenyl)acetic acid
	3-Methoxyphenylethanoic acid		(LEGPZHPSIPPYIO-UHFFFAOYSA-N;166.062994 g/mol)
4′-Hydroxyphenylacetic acid	4-Hydroxyphenylacetic acid^8^	156-38-7	2-(4-Hydroxyphenyl)acetic acid
	4-Hydroxyphenylethanoic acid		(XQXPVVBIMDBYFF-UHFFFAOYSA-N;152.047344 g/mol)
3′,4′-Dihydroxyphenylacetic acid	3,4-Dihydroxyphenylacetic acid^8^	102-32-9	2-(3,4-Dihydroxyphenyl)acetic acid
	3,4-Dihydroxyphenylethanoic acid		(CFFZDZCDUFSOFZ-UHFFFAOYSA-N;
	Homoprotocatechuic acid		168.042259 g/mol)
	DOPAC		
4′-Hydroxy-3′-methoxyphenylacetic acid	4-Hydroxy-3-methoxyphenylacetic acid^8^	306-08-1	2-(4-Hydroxy-3-methoxyphenyl)acetic acid
	4-Hydroxy-3-methoxyphanylethanoic acid		(QRMZSPFSDQBLIX-UHFFFAOYSA-N;
	Homovanillic acid		182.057909 g/mol)
8. Hydroxy-2-(phenyl)acetic acids (C_6_–C_2_)^9^			
2-Hydroxy-2-(3′-hydroxyphenyl)acetic acid	2-Hydroxy-2-(3-hydroxyphenyl)acetic acid^8^	17119-15-2	2-Hydroxy-2-(3-hydroxyphenyl)acetic acid
	(3-Hydroxyphenyl)-2-hydroxyacetic acid		(OLSDAJRAVOVKLG-UHFFFAOYSA-N;
	2-Hydroxy-2-(3-hydroxyphenyl)ethanoic acid		168.042259 g/mol)
	(3-Hydroxyphenyl)-2-hydroxyethanoic acid		
	3-Hydroxymandelic acid		
2-Hydroxy-2-(4′-hydroxyphenyl)acetic acid	2-Hydroxy-2-(4-hydroxyphenyl)acetic acid^8^	1198-84-1	2-Hydroxy-2-(4-hydroxyphenyl)acetic acid
	(4-Hydroxyphenyl)-2-hydroxyacetic acid		(YHXHKYRQLYQUIH-UHFFFAOYSA-N;
	2-Hydroxy-2-(4′-hydroxyphenyl)ethanoic acid		168.042259 g/mol)
	(4-Hydroxyphenyl)-2-hydroxyethanoic acid		
	4′-Hydroxymandelic acid		
	4-Hydroxymandelic acid		
	4-Hydroxy-d-mandelic acid		
	4-Hydroxy-l-mandelic acid		
2-Hydroxy-2-(4′-hydroxy-3′-methoxyphenyl)acetic acid	2-Hydroxy-2-(4-hydroxy-3-methoxyphenyl)acetic acid^8^	2394-20-9,	2-Hydroxy-2-(4-hydroxy-3-methoxyphenyl)acetic acid
	(4-Hydroxy-3-methoxyphenyl)-2-hydroxyacetic acid	55-10-7	(CGQCWMIAEPEHNQ-UHFFFAOYSA-N;
	2-Hydroxy-2-(4-hydroxy-3-methoxyphenyl)ethanoic acid		198.052823 g/mol)
	(4-Hydroxy-3-methoxyphenyl)-2-hydroxyethanoic acid		
	4′-Hydroxy-3′-methoxymandelic acid		
	4-Hydroxy-3-methoxymandelic acid		
	3-Methoxy-4-hydroxymandelic acid		
	4-Hydroxy-3-methoxy-l-mandelic acid		
	Vanillylmandelic acid		
9. Hippuric acids (C_6_–C_2_–N)			
Hippuric acid	—	495-69-2	2-Benzamidoacetic acid (QIAFMBKCNZACKA-UHFFFAOYSA-N;179.058243 g/mol)
2′-Hydroxyhippuric acid	2-Hydroxyhippuric acid^8^	487-54-7	2-[(2-Hydroxybenzoyl)amino]acetic acid
	2-(2-Hydroxybenzamido)acetic acid		(ONJSZLXSECQROL-UHFFFAOYSA-N;
	*N*-(2-hydroxybenzoyl)glycine		195.053158 g/mol)
	Salicyluric acid		
3′-Hydroxyhippuric acid	3-Hydroxyhippuric acid^8^	1637-75-8	2-[(3-Hydroxybenzoyl)amino]acetic acid
	2-(3-Hydroxybenzamido)acetic acid		(XDOFWFNMYJRHEW-UHFFFAOYSA-N;
	*N*-(3-hydroxybenzoyl)glycine		195.053158 g/mol)
4′-Hydroxhippuric acid	4-Hydroxyhippuric acid^8^	2482-25-9	2-[(4-Hydroxybenzoyl)amino]acetic acid
	2-(4-Hydroxybenzamido)acetic acid		(ZMHLUFWWWPBTIU-UHFFFAOYSA-N;
	*N*-(4-hydroxybenzoyl)glycine		195.053158 g/mol)
4′-Methoxyhippuric acid	4-Methoxyhippuric acid^8^	—	2-[(4-Methoxybenzoyl)amino]acetic acid
	2-(4-Ethoxybenzamido)acetic acid		(SIEIOUWSTGWJGE-UHFFFAOYSA-N;
	*N*-(4-methoxybenzoyl)glycine		209.068808 g/mol)
10. Phenyl-γ-valerolactones (C_6_–C_5_)^9,10^			
5-(3′,4′-Dihydroxyphenyl)-γ-valerolactone	5-(3,4-Dihydroxyphenyl)-γ-valerolactone^8^	21618-92-8	5-[(3,4-Dihydroxyphenyl)methyl]oxolan-2-one
	δ-(3,4-Dihydroxyphenyl)-γ-valerolactone5-(Dihydroxyphenyl)-γ-valerolactone	1108192-01-3 (4*S*)	(ZNXXWTPQHVLMQT-UHFFFAOYSA-N; 208.073559 g/mol)^9^
	5-(Dihydroxyphenyl)-valerolactone	191666-22-5	
	[*M6*]	(4*R*)	
5-(4′-Hydroxyphenyl)-γ-valerolactone-3′-glucuronide	5-(4-Hydroxyphenyl)-γ-valerolactone-3-glucuronide[*M6-glucuronide*][*5-(3′,4′-Dihydroxyphenyl)-γ-valerolactone-3'-glucuronide*]	—	3,4,5-Trihydroxy-6-[2-hydroxy-5-[(5-oxooxolan-2-yl)methyl]phenoxy]oxane-2-carboxylic acid (UVGDTVGPBRLMQY-UHFFFAOYSA-N;384.105647 g/mol)
5-(3′-Hydroxyphenyl)-γ-valerolactone-4′-glucuronide	5-(3-Hydroxyphenyl)-γ-valerolactone-4-glucuronide^8^[*M6-glucuronide*][*5-(3′,4′-Dihydroxyphenyl)-γ-valerolactone-4′-glucuronide*]	—	3,4,5-Trihydroxy-6-{2-hydroxy-4-[(5-oxooxolan-2-yl)methyl]phenoxy}oxane-2-carboxylic acid (OTBJYBQGMPICIK-UHFFFAOYSA-N;384.105647 g/mol)^9^
5-(4′-Hydroxyphenyl)-γ-valerolactone-3′-sulfate	5-(4-Hydroxyphenyl)-γ-valerolactone-3-sulfate^8^[*M6-sulfate*]	—	[2-Hydroxy-5-[(5-oxooxolan-2-yl)methyl]phenyl] hydrogen sulfate
	[*5-(3′,4′-Dihydroxyphenyl)-γ-valerolactone-3′-sulfate*]		(YAXFVDUJDAQPTJ-UHFFFAOYSA-N; 288.030374 g/mol)
5-(3′-Hydroxyphenyl)-γ-valerolactone-4′-sulfate	5-(3-Hydroxyphenyl)-γ-valerolactone-4-sulfate^8^[*M6-sulfate*]	—	[2-Hydroxy-4-[(5-oxooxolan-2-yl)methyl]phenyl] hydrogen sulfate
	[*5-(3′,4′-Dihydroxyphenyl)-γ-valerolactone-4′-sulfate*]		(WAXYAOJFDCCESK-UHFFFAOYSA-N; 288.030374 g/mol)
5-(3′-Methoxyphenyl)-γ-valerolactone-4′-sulfate	5-(3-Methoxyphenyl)-γ-valerolactone-4-sulfate^8^[*Methyl-M6-sulfate*]	—	[2-Methoxy-4-[(5-oxooxolan-2-yl)methyl]phenyl] hydrogen sulfate
	[*5-(4′-Hydroxy-3′-methoxyphenyl)-γ-valerolactone-4′-sulfate*]		(FYRRHCSCZYSADR-UHFFFAOYSA-N;
	[*5-(3′,4′-Dihydroxyphenyl)-γ-valerolactone-3′-methoxy-4′-sulfate*]		302.046024 g/mol)
5-(3′-Methoxyphenyl)-γ-valerolactone-4′-glucuronide	5-(3-Methoxyphenyl)-γ-valerolactone-4-glucuronide^8^[*Methyl-M6-glucuronide*][*5-(4′-Hydroxy-3′-methoxyphenyl)-γ-valerolactone-4′-glucuronide*][*5-(3′,4′-Dihydroxyphenyl)-γ-valerolactone-3'-methoxy-4'-glucuronide*]	—	3,4,5-Trihydroxy-6-[2-methoxy-4-[(5-oxooxolan-2-yl)methyl]phenoxy]oxane-2-carboxylic acid (NGMVEYPQYAIGEI-UHFFFAOYSA-N; 398.121297 g/mol)
5-(Phenyl)-γ-valerolactone-3′-sulfate-4′-glucuronide	5-(Phenyl)-γ-valerolactone-3-sulfate-4-glucuronide^8^	—	—
	[*M6-sulfate-glucuronide*]		
	[5-(*3′,4′-Dihydroxyphenyl*)-γ-valerolactone-3′*-sulfate-4′-glucuronide*]		
11. 4-Hydroxy-5-(phenyl)valeric acids (C_6_–C_5_)^9^			
4-Hydroxy-5-(3′,4′-dihydroxyphenyl)valeric acid	4-Hydroxy-5-(3,4-dihydroxyphenyl)valeric acid^8^5-(3,4-Dihydroxyphenyl)-4-hydroxyvaleric acid4-Hydroxy-5-(3,4-dihydroxyphenyl)pentanoic acid	—	5-(3,4-Dihydroxyphenyl)-4-hydroxypentanoic acid (JDBYFCLHVYVXCX-UHFFFAOYSA-N; 226.084124 g/mol)
	5-(3,4-Dihydroxyphenyl)-4-hydroxypentanoic acid		
	[*5-(3,4-Dihydroxyphenyl)-γ-hydroxyvaleric acid*]		
	[*5-(3,4-Dihydroxyphenyl)-γ-hydroxypentanoic acid*]		
4-Hydroxy-5-(4′-hydroxyphenyl)valeric acid-3′-glucuronide	4-Hydroxy-5-(4-hydroxyphenyl)valeric acid-3-glucuronide^8^5-(4-Hydroxyphenyl)-4-hydroxyvaleric acid-3-glucuronide4-Hydroxy-5-(4-hydroxyphenyl)pentanoic acid-3-glucuronide	—	6-[5-(4-Carboxy-2-hydroxybutyl)-2-hydroxyphenoxy]-3,4,5-trihydroxyoxane-2-carboxylic acid (BKJQCPDRWDNBIN-UHFFFAOYSA-N;
	5-(4-Hydroxyphenyl)-4-hydroxypentanoic acid-3-glucuronide		402.116212 g/mol)
	[*5-(3,4-Dihydroxyphenyl-4-hydroxyvaleric acid-3-glucuronide*]		
4-Hydroxy-5-(3′-hydroxyphenyl)valeric acid-4′-glucuronide	4-Hydroxy-5-(3-hydroxyphenyl)valeric acid-4-glucuronide^8^5-(3-Hydroxyphenyl)-4-hydroxyvaleric acid-4-glucuronide4-Hydroxy-5-(3-hydroxyphenyl)pentanoic acid-4-glucuronide	—	6-[4-(4-Carboxy-2-hydroxybutyl)-2-hydroxyphenoxy]-3,4,5-trihydroxyoxane-2-carboxylic acid (LLKUARGHNZMSRT-UHFFFAOYSA-N;
	5-(4-Hydroxyphenyl)-4-hydroxyvaleric acid-4-glucuronide		402.116212 g/mol)
	[*5-(3′,4'′Dihydroxyphenyl)-4-hydroxyvaleric acid-4′-glucuronide*]		
4-Hydroxy-5-(4′-hydroxyphenyl)valeric acid-3′-sulfate	4-Hydroxy-5-(4-hydroxyphenyl)valeric acid-3-sulfate^8^5-(4-Hydroxyphenyl)-4-hydroxyvaleric acid-3-sulfate4-Hydroxy-5-(4-hydroxyphenyl)pentanoic acid-3-sulfate	—	4-Hydroxy-5-(4-hydroxy-3-sulfooxyphenyl)pentanoic acid (HROSNTXKMPHTSL-UHFFFAOYSA-N; 306.040939 g/mol)
	5-(4-Hydroxyphenyl)-4-hydroxypentanoic acid-3-sulfate		
	[*5-(3′,4′-Dihydroxyphenyl)-4-hydroxyvaleric acid-3′-sulfate*]		
4-Hydroxy-5-(3′-hydroxyphenyl)valeric acid-4′-sulfate	4-Hydroxy-5-(3-hydroxyphenyl)valeric acid-4-sulfate^8^	—	—
	5-(3-Hydroxyphenyl)-4-hydroxyvaleric acid-4-sulfate		
	4-Hydroxy-5-(3-hydroxyphenyl)pentanoic acid-4-sulfate		
	5-(3-Hydroxyphenyl)-4-hydroxypentanoic acid-4-sulfate		
	[*5-(3′,4′-Dihydroxyphenyl)-4-hydroxyvaleric acid-4′-sulfate*]		
4-Sulfoxy-5-(3′,4′-dihydroxyphenyl)valeric acid	4-Sulfoxy-5-(3,4-dihydroxyphenyl)valeric acid^8^	—	5-(3,4-Dihydroxyphenyl)-4-sulfooxypentanoic acid
	5-(3,4-Dihydroxyphenyl)-4-sulfoxyvaleric acid		(NBKHZVHBUVNGQF-UHFFFAOYSA-N;
	4-Sulfoxy-5-(3,4-dihydroxyphenyl)pentanoic acid		306.040939 g/mol)
	5-(3,4-Dihydroxyphenyl)-4-sulfoxypentanoic acid		
	[*5-(3-Hydroxyphenyl)-4-sulphoxyvaleric acid*]		
4-Hydroxy-5-(3′-methoxyphenyl)valeric acid-4′-sulfate	4-Hydroxy-5-(3-methoxyphenyl)valeric acid-4-sulfate^8^	—	4-Hydroxy-5-[3-methoxy-4-(sulfooxy)phenyl]pentanoic acid
	5-(3-Methoxyphenyl)-4-hydroxyvaleric acid-4-sulfate		(GUEAZORXKKSCMA-UHFFFAOYSA-N;
	4-Hydroxy-5-(3-methoxyphenyl)pentanoic acid-4-sulfate		320.056589 g/mol)
	5-(3-Methoxyphenyl)-4-hydroxypentanoic acid-4-sulfate		
	[*4-Hydroxy-5-(3,4-dihydroxyphenyl)-valeric acid-3-methoxy-4-sulfate*]		
4-Hydroxy-5-(4′-methoxyphenyl)valeric acid-3′-sulfate	4-Hydroxy-5-(4-methoxyphenyl)valeric acid-3-sulfate^8^	—	—
	5-(4-Methoxyphenyl)-4-hydroxyvaleric acid-3-sulfate		
	4-Hydroxy-5-(4′-methoxyphenyl)pentanoic acid-3′-sulfate		
	5-(4-Methoxyphenyl)-4-hydroxpentanoic acid-3-sulfate		
	[*4-Hydroxy-5-(3,4-dihydroxyphenyl)-valeric acid-3-methoxy-4-sulfate*]		
4-Sulfoxy-5-(4′-hydroxy-3′-methoxyphenyl)valeric acid	4-Sulfoxy-5-(4-hydroxy-3-methoxyphenyl)valeric acid^8^	—	5-(4-Hydroxy-3-methoxyphenyl)-4-(sulfooxy)pentanoic acid
	5-(4-Hydroxy-3-methoxyphenyl)-4-sulfoxyvaleric acid		(JQISKEAFUDWLCF-UHFFFAOYSA-N;
	4-Sulfoxy-5-(4-hydroxy-3-methoxyphenyl)pentanoic acid		320.056589 g/mol)
	5-(4-Hydroxy-3-methoxyphenyl)-4-sulfoxypentanoic acid		
	[*4-Hydroxy-5-(3,4-dihydroxyphenyl)-valeric acid-3-methoxy-4-sulfate*]		
4-Hydroxy-5-(3′-methoxyphenyl)valeric acid-4′-glucuronide	4-Hydroxy-5-(3-methoxyphenyl)valeric acid-4- glucuronide^8^5-(3-Methoxyphenyl)-4-hydroxyvaleric acid-4-glucuronide4-Hydroxy-5-(3-methoxyphenyl)pentanoic acid-4-glucuronide	—	6-[4-(4-Carboxy-2-hydroxybutyl)-2-methoxyphenoxy]-3,4,5-trihydroxyoxane-2-carboxylic acid (WOBDOUCWWPCYBL-UHFFFAOYSA-N;
	5-(3′-Methoxyphenyl)-4-hydroxypentanoic acid-4-glucuronide		416.131862 g/mol)
	[*4-Hydroxy-5-(3′,4′-dihydroxyphenyl)-valeric acid-3′-methoxy-4′-glucuronide*]		
12. 5-Phenylvaleric acids (C_6_–C_5_)			
5-(3′,5′-Dihydroxyphenyl)valeric acid	5-(3,5-Dihydroxyphenyl)valeric acid^8^	74356-41-5	5-(3,5-Dihydroxyphenyl)pentanoic acid
	5-(3,5-Dihydroxyphenyl)pentanoic acid		(QHXNJIMVPAFCPR-UHFFFAOYSA-N;
	[*5-(Dihydroxyphenyl)valeric acid*]		210.089203 g/mol)
	[*Dihydroxy-benzenepentanoic acid*]		
5-(3′,4′,5′-Trihydroxyphenyl)valeric acid	5-(3,4,5-Trihydroxyphenyl)valeric acid^8^	—	5-(3,4,5-Trihydroxyphenyl)pentanoic acid
	5-(3,4,5-Trihydroxyphenyl)pentanoic acid		(MASHJJSDLKLZLA-UHFFFAOYSA-N;
	[*5-(Trihydroxyphenyl)valeric acid*]		226.084124 g/mol)
	[*Trihydroxy-benzenepentanoic acid*]		
13. Urolithins (6H-Dibenzo[b,d]pyran-6-one)			
3-Hydroxy-urolithin	Urolithin B	1139-83-9	3-Hydroxy-6H-benzo[c]chromen-6-one (WXUQMTRHPNOXBV-UHFFFAOYSA-N; 212.047344 g/mol)
Urolithin-3-glucuronide	Urolithin B-3-glucuronide[*Urolithin B-glucuronide*]	823806-74-2	(2*S*,3*S*,4*S*,5*R*,6*S*)-3,4,5-trihydroxy-6-({6-oxo-6H-benzo[c]chromen-3-yl}oxy)oxane-2-carboxylic acid (MHBWCULXQBVPQT-KSPMYQCISA-N; 388.079432 g/mol)
Urolithin-3-sulfate	Urolithin B-3-sulfate	—	(6-Oxobenzo[c]chromen-3-yl) hydrogen sulfate
	[*Urolithin B-sulfate*]		(LRRVKQQFGIEMSA-UHFFFAOYSA-N; 292.004159 g/mol)
3,8-Dihydroxy-urolithin	Urolithin A	1143-70-0	3,8-Dihydroxy-6H-benzo[c]chromen-6-one (RIUPLDUFZCXCHM-UHFFFAOYSA-N; 228.042259 g/mol)
8-Hydroxy-urolithin-3-glucuronide	Urolithin A-3-glucuronide[*Urolithin A-glucuronide*][*3,8-Dihydroxy-urolithin 3-glucuronide*]	—	3,4,5-Trihydroxy-6-(8-hydroxy-6-oxo-6H-benzo[c]chromen-3-yl)oxyoxane-2-carboxylic acid (KXBXNRJGUDTJQS-UHFFFAOYSA-N; 404.074347 g/mol)
3-Hydroxy-urolithin-8-glucuronide	Urolithin A-8-glucuronide[*Urolithin A-glucuronide*][*3,8-Dihydroxy-urolithin 8-glucuronide*]	1365982-52-0	(2*S*,3*S*,4*S*,5*R*,6*S*)-3,4,5-trihydroxy-6-(3-hydroxy-6-oxobenzo[c]chromen-8-yl)oxyoxane-2-carboxylic acid (QMPHAAMUHRNZSL-KSPMYQCISA-N; 404.074347 g/mol)
Urolithin-3,8-diglucuronide	Urolithin A-3,8-diglucuronide[*Urolithin A-diglucuronide*][*3,8-Dihydroxy-urolithin-diglucuronide*]	—	(2*S*,3*S*,4*S*,5*R*,6*S*)-6-[3-[(2*S*,3*R*,4*S*,5*S*,6*S*)-6-carboxy-3,4,5-trihydroxyoxan-2-yl]oxy-6-oxobenzo[c]chromen-8-yl]oxy-3,4,5-trihydroxyoxane-2-carboxylic acid(SXMJSEFKPOZNAT-ILJCXFEASA-N; 580.106435 g/mol)
8-Hydroxy-urolithin-3-sulfate	Urolithin A-3-sulfate	—	(8-Hydroxy-6-oxobenzo[c]chromen-3-yl) hydrogen sulfate
	[*Urolithin A-sulfate*]		(WMPNAWQWWZFJTQ-UHFFFAOYSA-N;
	[*3,8-Dihydroxy-urolithin 3-sulfate*]		307.999074 g/mol)
3-Hydroxy-urolithin-8-sulfate	Urolithin A-8-sulfate[*Urolithin A-sulfate*][*3,8-Dihydroxy-urolithin 8-sulfate*]		(3-Hydroxy-6-oxo-6H-benzo[c]chromen-8-yl)oxidanesulfonic acid
Urolithin-3-sulfate-8-glucuronide	[*Urolithin A-sulfate-glucuronide*]	—	—
	[*3,8-Dihydroxy-urolithin 3-sulfate-8-glucuronide*]		
3,9-Dihydroxy-urolithin	Isourolithin A	174023-48-4	3,9-Dihydroxy-6H-benzo[c]chromen-6-one (WDGSXHQNUPZEHA-UHFFFAOYSA-N; 228.042259 g/mol)
9-Hydroxy-urolithin-3-glucuronide	Isourolithin A-3-glucuronide	—	—
	[*Isourolithin A-glucuronide*]		
	[*3,9-Dihydroxy-urolithin-3-glucuronide*]		
3-Hydroxy-urolithin-9-glucuronide	Isourolithin A-9-glucuronide	—	—
	[*Isourolithin A-glucuronide*]		
	[*3,9-Dihydroxy-urolithin 9-glucuronide*]		
3,8,9-Trihydroxy-urolithin	Urolithin C	165393-06-6	3,8,9-Trihydroxy-6H-benzo[c]chromen-6-one
	[*Hydroxyurolithin A*]		(HHXMEXZVPJFAIJ-UHFFFAOYSA-N;
	[*Trihydroxyurolithin*]		244.037173 g/mol)
8,9-Dihydroxy-urolithin-3-glucuronide	Urolithin C-3-glucuronide[*Urolithin C-glucuronide*]	1268248-76-5	(2*S*,3*S*,4*S*,5*R*,6*S*)-6-({8,9-dihydroxy-6-oxo-6H-benzo[c]chromen-3-yl}oxy)-3,4,5-trihydroxyoxane-2-carboxylic acid (DDAQYQCCOWZGDO-KSPMYQCISA-N; 420.069261 g/mol)
3,4,8,9-Tetrahydroxy-urolithin	Urolithin D	131086-98-1	3,4,8,9-Tetrahydroxy-6H-benzo[c]chromen-6-one (NEZDQSKPNPRYAW-UHFFFAOYSA-N; 260.032088 g/mol)
3,8,9,10-Tetrahydroxy-urolithin	Urolithin M6	1006683-97-1	3,8,9,10-Tetrahydroxy-6H-benzo[c]chromen-6-one (LGXFTZDSEIQMMP-UHFFFAOYSA-N; 260.032088 g/mol)
3,8,10-Trihydroxy-urolithin	Urolithin M7	531512-26-2	3,8,10-Trihydroxy-6H-benzo[c]chromen-6-one (AKJHSPSPAOUDFT-UHFFFAOYSA-N; 244.037173 g/mol)
14. Others (C_6_–C_2_)			
2-(3′-Hydroxyphenyl)ethanol	2-(3-Hydroxyphenyl)ethanol^8^	10597-60-1	4-(2-Hydroxyethyl)-1,2-benzenediol
	3-Hydroxytyrosol		(JUUBCHWRXWPFFH-UHFFFAOYSA-N;
	3,4-Dihydroxyphenylethanol		154.062994 g/mol)
	3,4-Dihydroxyphenethyl alcohol		
2-(3′,4′-Dihydroxyphenyl)ethanol	4-Hydroxyphenethyl alcohol^8^Tyrosol	501-94-0	4-(2-Hydroxyethyl)phenol (YCCILVSKPBXVIP-UHFFFAOYSA-N; 138.06808 g/mol)

1 This table was established based on cross-referencing the following databases: PubChem (https://pubchem.ncbi.nlm.nih.gov), NIST Chemistry WebBook (https://webbook.nist.gov), ChemIDPlus (https://chem.nlm.nih.gov/chemidplus), HMDB (http://www.hmdb.ca), ChemSpider (http://www.chemspider.com), ChemicalBook (https://www.chemicalbook.com), and ChEMBL (https://www.ebi.ac.uk). Column 1, proposed nomenclature; column 2, equally accurate alternative “nonprime” nomenclature indicated by superscript 8, followed by common or trivial names, and, finally, often reported inaccurate nomenclature shown in italics and brackets. Table 1 is available for download (Excel file; **Supplemental Table 1**). CAS-RN, Chemical Abstracts Service Registry Number; DOPAC, 3′,4′-dihydroxyphenylacetic acid; IUPAC, International Union of Pure and Applied Chemistry.

2 Glucuronic acid–oxygen conjugations are of β*-*d*-*configuration.

3 Column 2 contains synonyms along with incorrect nomenclature that are in brackets. Both should be avoided to prevent further confusion in the literature and in online databases.

4 CAS Registry Number (CAS-RN) is a numeric identifier that can contain ≤10 digits, divided by hyphens into 3 parts, and each number is a unique numeric identifier that can link to information about the substance to which it refers, but it has no chemical significance per se (see https://www.cas.org/support/documentation/chemical-substances). Not all compounds have a CAS number, typically because they have a limited or no commercial source.

5 The IUPAC is described as the world authority on chemical nomenclature and terminology (https://iupac.org/who-we-are).

6 InChIKey: IUPAC standard textual unique chemical identifier.

7 Cinnamic acids have *cis* (*Z*) and *tran*s (*E*) geometric isomers. In nature, the *trans* isomer is more common.

8 Alternative “nonprime” nomenclatre.

9 Compounds that occur as *R*- and *S*-isomers.

10 In rare instances, compounds appear to have 2 different InChIKey formulas in online databases which are generally associated with different CAS or registry numbers and possibly reflect uncharacterized isomeric configuration. For example, 5-(3′,4′-dihydroxyphenyl)-γ-valerolactone or IUPAC 5-[(3,4-dihydroxyphenyl)methyl]oxolan-2-one is listed as having 2 different CAS RNs: 21618-92-8 and 191666-22-5; 2 different standard 27-character InchiKeys: ZNXXWTPQHVLMQT-UHFFFAOYSA-N and ZNXXWTPQHVLMQT-MRVPVSSYSA-N; and sharing the same simplified molecular-input line-entry system (SMILES) formula: C1CC(=O)OC1CC2=CC(=C(C=C2)O)O. In this case, each structure has a unique full InChI (using the SHA-256 algorithm): InChI=1S/C11H12O4/c12-9-3-1-7(6-10(9)13)5-8-2-4-11(14)15-8/h1,3,6,8,12-13H,2,4-5H2 and 1S/C11H12O4/c12-9-3-1-7(6-10(9)13)5-8-2-4-11(14)15-8/h1,3,6,8,12-13H,2,4-5H2/t8-/m1/s1, which should be used in cases in which 2 isomers require distinction. Similarly, 5-(3′-hydroxyphenyl)-γ-valerolactone-4′-glucuronide or IUPAC 3,4,5-trihydroxy-6-{2-hydroxy-4-[(5-oxooxolan-2-yl)methyl]phenoxy}oxane-2-carboxylic acid has two different standard InChIKeys—OTBJYBQGMPICIK-UHFFFAOYSA-N and OTBJYBQGMPICIK-GHPVWUPISA-N—and different simplified molecular-input line-entry system (SMILES) formula and full InChIKey.

Biological activity, or its absence, is a function of the 3D structure of a molecule. The goal of a nomenclature system is to describe this 3D structure unambiguously allowing translation of text to a 2D illustrated structure, in a manner as accurate as the available experimental data allow. It should be easy to use and understand, and accommodate multiple forms of isomerism, to permit distinction of isomeric structures with ease. There is, however, no perfect system capable of achieving this objective in a convenient manner that also accommodates all compounds in all situations. Accordingly, this article focuses on the commonly reported isomeric forms of (poly)phenol catabolites and their metabolites, recommending a system of nomenclature and also providing a thesaurus to facilitate translation between different systems of nomenclature. This is important because with many compounds, five and as many as ten synonyms and styles of nomenclature, some incorrect, appear in the nutrition literature (Table 1). In the proposed nomenclature, IUPAC-based names are used, but in instances in which IUPAC terminology is inconsistent (e.g., phenylpropanoic and phenylpropionic acids) or is overly complex and/or where alternative names have become accepted and widely used (e.g., hippuric acids, phenyl-γ-valerolactones, phenylvaleric acids, and urolithins), modifications are used.

The phenolic compounds that are covered in Table 1 are those originating from acyl-quinic acids, ellagitannins, and the main dietary flavonoids—namely anthocyanins, flavones, flavonols, flavanones, and flavan-3-ols—as well as the avenanthramides and alkyl-resorcinols characteristic of the staple foods wheat, rye, and quinoa. It does not cover catabolites of minor dietary (poly)phenols or those with a limited distribution, such as isoflavones, stilbenes, phenylpropenes, lignans, and iridoids (oleuropeins). Catabolites of these (poly)phenols also require a standardized nomenclature, which is beyond the scope of this article.

## Nomenclature of Phenolic Catabolites

**FIGURE 1:**
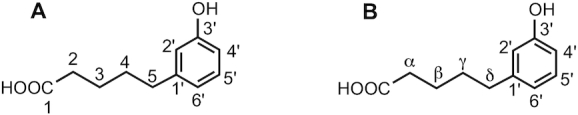
Numbering of the phenolic ring and its side chain with the current practice (A) and the older less fashionable use of lowercase Greek letters for the side chain (B).

(Poly)phenolic catabolites found in blood, tissues, urine, and feces can be easily subdivided using a shorthand system to describe their molecular skeleton by concisely defining the number of carbon atoms on the phenyl ring and its side chain and whether the side chain is unsaturated (i.e., containing double bonds) and/or contains nitrogen or substituents such as a hydroxyl group. In this shorthand system, C_6_ identifies a phenyl ring with its 6 carbon atoms (**Figure 1**), and the number of carbon atoms in the side chain is defined using C_0_, C_1_, C_2_, etc., as necessary—that is, C_6_–C_0_, C_6_–C_1_, C_6_–C_2_, etc. The most extensively studied catabolites have a side chain with a terminal carboxyl group that is designated C-1. Further description of the molecule becomes more complicated because the literature contains trivial names, some of which are synonyms, such as acetic acid and ethanoic acid. For a C_6_−C_3_ structure, the synonyms cinnamic, propenoic, and acrylic all signify an unsaturated C_3_ side chain with a double bond between C-2 and C-3. The synonyms propionic, propanoic, and dihydrocinnamic acid denote a saturated C_3_ side chain. Longer side chains include pentanoic (C_6_–C_5_), also described as valeric, and the less common heptanoic (C_6_–C_7_) and nonanoic (C_6_–C_9_). The term phenyl-pentenoic identifies a C_6_–C_5_ catabolite with an unsaturated side chain but does not specify the location of the double bond. This can be defined by identifying the lowest-number carbon associated with it—for example, pent-2-enoic or pent-3-enoic—and, if possible, specifying whether it is of a *cis* (*Z*) or *trans* (*E*) configuration. The current practice for numbering the carbon atoms in these compounds is shown in Figure 1A, with the former less fashionable practice of using lowercase Greek letters shown in Figure 1B, and illustrates the proposed framework for simplifying phenolic metabolite nomenclature.

Figure 1 also illustrates how the ring carbons can be distinguished from the side-chain carbons using a number bearing a prime—for example, 3′—rather than simply 3. This use of a prime is of value when otherwise a structure would contain two atoms that might reasonably be described as “3,” as in 3-(3′-hydroxyphenyl)propanoic acid, in which 3 refers to the number of carbons in the side chain (counting from the carboxyl carbon, COOH = 1) and 3′ refers to the position of substituent groups on the phenyl ring, with 1′ being the carbon on the phenyl ring bearing the primary side chain. This use of prime numbers may not be essential by IUPAC convention, and seems superfluous to seasoned chemists, but it does not require an extensive understanding of nomenclature to facilitate its use. The system recommended here is designed to be easily accessible to nonchemists.

**FIGURE 2:**
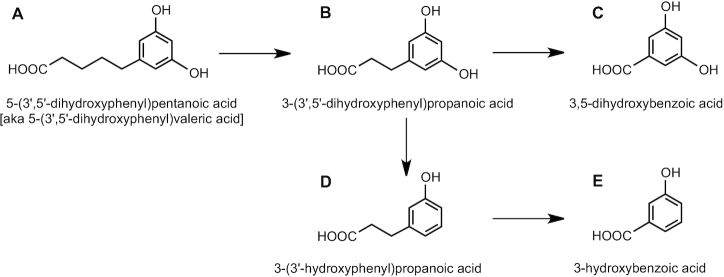
Nomenclature of metabolites involved in the microbial conversion of C_6_–C_5_ to C_6_–C_3_ and C_6_–C_1_.

**FIGURE 3:**
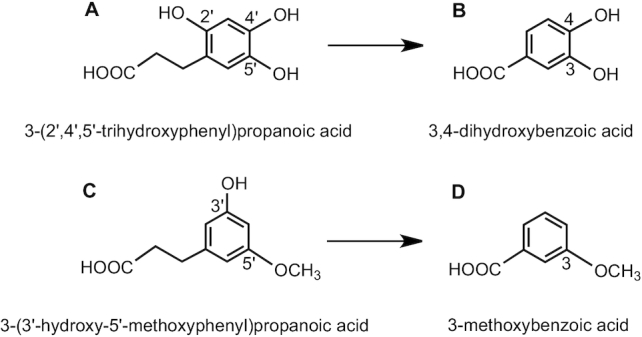
An illustration of how numbering of substituents may change during catabolism.

For C_6_–C_0_ or C_6_–C_1_ structures such as phenols and benzoic acids, the use of prime numbers is not required because the phenyl ring is the only site for substituent groups. This is illustrated in **Figure 2** with the conversion of 3-(3′,5′-dihydroxyphenyl)propanoic acid (Figure 2B) to 3,5-dihydroxybenzoic acid (Figure 2C) and 3-hydroxybenzoic acid (Figure 2E).

The advantage of the prime symbol is clear with a structure such as 3-(3′-hydroxyphenyl)propanoic acid, but one could argue it is not entirely necessary to use a prime number for 3-(4′-hydroxyphenyl)propanoic acid because there are only 3 carbons in the side chain and no risk of assigning a bond position twice. However, this can become confusing when there are ≥2 substituents in the ring, as in 3-(3′,4′-dihydroxyphenyl)propanoic acid, or if 1 of the hydroxyls in the phenolic ring is a biological conjugate, as in 3-(3′-hydroxyphenyl)propanoic acid-4′-sulfate, a feature discussed more extensively later. Table 1 outlines the proposed and alternatively accepted nomenclature for reference and for integration of published works and databases. Examples of the proposed nomenclature are provided in column 1 of Table 1, with equally accurate but alternative “nonprime” nomenclature, often found in online databases, presented in column 2 (superscript 8), followed by common or trivial names, and, finally, in italics and brackets, the often reported nomenclature for phase II conjugates that is inaccurate because of the double assigning of carbon atoms, as further discussed later. The use of trivial names and inaccurate nomenclature should be avoided to prevent further confusion in the literature and online databases. Column 3 contains CAS numbers (https://www.cas.org/support/documentation/chemical-substances), and column 4 includes the IUPAC nomenclature followed in parentheses by standard InChIKey chemical identifiers (https://iupac.org/who-we-are) and monoisotopic mass values.

**Figure 3** illustrates how the numbering of the ring carbons might change following metabolism. For example, when the 2′-OH is removed from the 3-(2′,4′,5′-trihydroxyphenyl)propanoic acid (Figure 3A) and the 3-carbon side chain is shortened, this produces 3,4-dihydroxybenzoic acid (Figure 3B) because in assigning numbers to the carbons bearing hydroxyl groups, it is necessary to take the shortest route from the carbon bearing the carboxyl moiety. This also applies to 3-(3′-hydroxy-5′-methoxyphenyl)propanoic acid (Figure 3C), in which the numbering of the hydroxyl is given priority over the methoxy group. When the hydroxyl is removed and the side chain shortened, the C_6_–C_1_ product is 3-methoxybenzoic acid (Figure 3D) rather than 5-methoxybenzoic acid.

The terms hydracrylic acid and lactic acid describe isomeric saturated C_3_ side chains bearing a hydroxyl, at C-3 for the hydracrylic acid side chain but at C-2 for a lactic acid side chain. These compounds are referred to as 3-hydroxy-3-(phenyl)propanoic acids and 2-hydroxy-3-(phenyl)propanoic acids, respectively. The alternative non-IUPAC names that are often used are 3-(phenyl)-3-hydroxypropionic and 2-hydroxy-3-(phenyl)propionic acids, respectively (Table 1, sections 6 and 8). For both of these types of compounds, there are *R-* and *S*-isomers determined by the orientation of the side-chain hydroxyl group.

**FIGURE 4:**
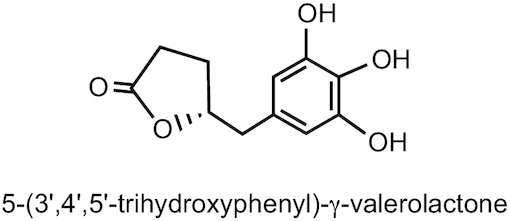
Structure of 5-(3′,4′,5′-trihydroxyphenyl)-γ-valerolactone.

Phenyl-γ-valerolactones, the main microbiota-derived metabolites of flavan-3-ols, are chemically related to 4-hydroxy-5-(phenyl)pentanoic acids. The most common species include 5-(3′,4′-dihydroxyphenyl)-γ-valerolactone, 5-(3′,5′-dihydroxyphenyl)-γ-valerolactone, and 5-(3′,4′,5′-trihydroxyphenyl)-γ-valerolactone (**Figure 4**). They have traditionally been abbreviated as M6, M6′, and M4, respectively, but this is now not recommended because it complicates accurate structural assignment. More details about standardized nomenclature for phenyl-γ-valerolactones can be found in a recent review article by Mena et al. (14).

**FIGURE 5:**
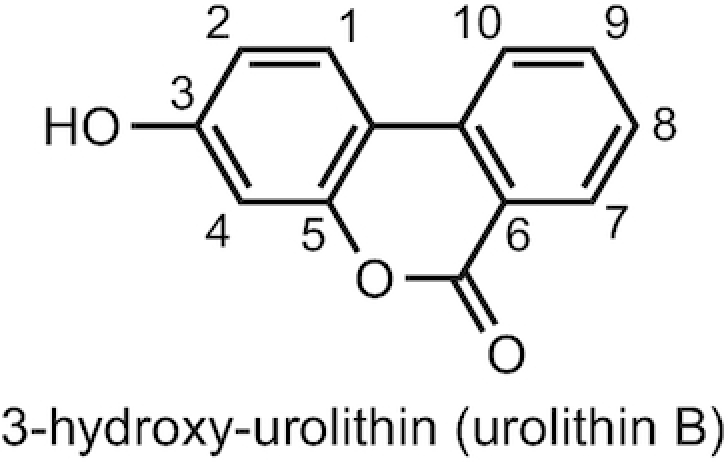
Urolithin nomenclature based on Gonzalez-Barrio et al. (15).

Urolithins, microbiota-mediated products derived from catabolism of ellagitannins, possess a 6H-dibenzo-[*b,d*]pyran-6-one structure (**Figure 5**), making them particularly difficult to assign appropriate structural nomenclature. They represent a special case because they possess 2 phenyl rings and lack a side chain, and therefore a slightly different numbering convention is required. The accepted system for numbering (15) is presented in Figure 5. The adoption of this system avoids issues associated with an oversimplified nomenclature often adopted in the literature, based on assigning “A,” “B,” or “C” to a structure, such as urolithin-A (3,8-dihydroxy-urolithin), -B (3-hydroxy-urolithin), and -C (3,8,9-trihydroxy-urolithin). The use of the A, B, C nomenclature is not recommended (Table 1) because it easily leads to inaccurate assignments, particularly when describing the positions of human phase II methyl, glucuronide, or sulfate conjugates.

## Phase II Metabolites of Phenolic Catabolites

The correct designation of mammalian phase II conjugates where a hydroxyl has been conjugated with a sulfate, glucuronide, or methyl group during the metabolism of dietary (poly)phenols, often causes confusion. For example, if 5-(3′,4′-dihydroxyphenyl)valeric acid is conjugated with a glucuronide moiety, the substrate effectively loses one hydroxyl and becomes 5-(3′-hydroxyphenyl)valeric acid-4′-glucuronide or 5-(4′-hydroxyphenyl)valeric acid-3′-glucuronide, depending on which hydroxyl is conjugated. It is essential to be able to distinguish such isomers. To use 5-(3′,4′-dihydroxyphenyl)valeric acid-3′-glucuronide would be inaccurate and is not recommended nomenclature because the 3′ position is assigned twice. However, in order to facilitate interpretation and translation of pre-existing publications and databases, such “double assignments” are shown, italicized and in brackets in column 2 of Table 1, because they are common in the literature. “Double assignment” is also encountered when catabolites are described using trivial names—for example, dihydrocaffeic acid, dihydrocaffeic acid-3-glucuronide, and dihydrocaffeic acid-4-glucuronide. This should also be avoided, and we recommend 3-(3′,4′-dihydroxyphenyl)propanoic acid, 3-(4′-hydroxyphenyl)propanoic acid-3′-glucuronide, and 3-(3′-hydroxyphenyl)propanoic acid-4′-glucuronide, respectively. Again, for the purposes of translation, trivial names are shown in column 2 of Table 1.

It is common when describing conjugated molecules to define the nature of the atom to which the conjugating moiety is attached—for example, *O*-glucuronide, *O*-sulfate, and *O*-methyl. This is necessary in any situation in which alternative points of attachment might be encountered, but as far as the authors are aware, there are no reported human glucuronide or sulfate metabolites of dietary (poly)phenols that are conjugated to a carbon atom. Accordingly, the use of “*O*” is redundant, albeit not incorrect, but use of the lowercase “*o*” is incorrect. We also recommend that methyl conjugates be referred to as “methoxy-” rather than “*O*-methyl” derivatives—for example, 3-(3′-hydroxy-4′-methoxyphenyl)propanoic acid.

The issue of conjugation is often further complicated by commercial vendors describing synthetic reference standards using inaccurate nomenclature. For example, many companies and online databases present the phase II metabolite of 3-(3′,4′-dihydroxyphenyl)propanoic acid as 3-(3′,4′-dihydroxyphenyl)propanoic acid-3′-glucuronide. As noted previously, a bond position for (poly)phenols and phenolic metabolites should not be assigned twice in structural nomenclature. The accurate representation for the structure under the proposed nomenclature is 3-(4′-hydroxyphenyl)propanoic acid-3′-glucuronide. As another example, phenyl-γ-valerolactone conjugates are often inaccurately annotated as 5-(3*′*,4′-dihydroxyphenyl)-γ-valerolactone-4′-glucuronide, although the correct nomenclature is 5-(3′-hydroxyphenyl)-γ-valerolactone-4′-glucuronide (14). The same applies to the naming of sulfate and methoxy metabolites. In addition, Greek symbols such as “α,” “β,” and “γ” may be presented as their textual equivalents “alpha,” “beta,” and “gamma” in some databases in which symbols may not be compatible with certain software or programs.

## Isomers

Dietary (poly)phenols, their catabolites, and phase II conjugates can display multiple forms of isomerism, and because biological activity is a function of the 3D structure, it is important to discriminate between isomers and describe their structure clearly and unambiguously. It is recommended that after determining the general nature of a metabolite, the number of possible isomers is calculated before assigning a more precise structure. The possible number may be surprisingly high, and if there are insufficient data to allow unequivocal discrimination between the possible structures, the description applied must make this clear. There may be good reasons to eliminate some of these possibilities and favor others, and this can be made clear in discussion, but overprecise assignment must be avoided. Previous detailed recommendations on this topic, which we endorse, were provided by Sumner et al. (16).

**FIGURE 6:**
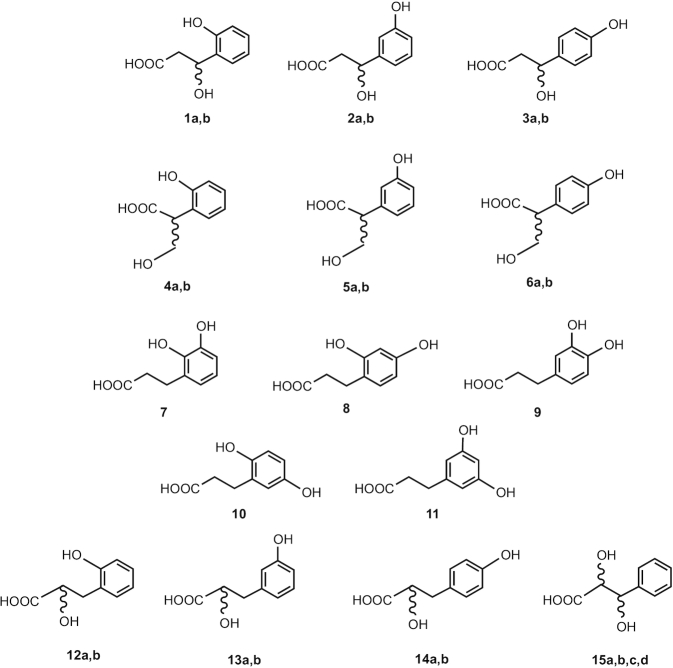
C_6_–C_3_ compounds with 2 hydroxyl groups and an exact mass of 182.0579 Da. 1a, b, 3*R/S*-hydroxy*-*3-(2′-hydroxyphenyl)propanoic acid; 2a, b, 3*R/S*-hydroxy*-*3-(3′-hydroxyphenyl)propanoic acid; 3a, b, 3*R/S*-hydroxy*-*3-(4′-hydroxyphenyl)propanoic acid; 4a, b, 2*R/S*-hydroxymethyl-2-(2′-hydroxyphenyl)acetic acid; 5a, b, 2*R/S*-hydroxymethyl-2-(3′-hydroxyphenyl)acetic acid; 6a, b, 2*R/S*-hydroxymethyl-2-(4′-hydroxyphenyl)acetic acid; 7, 3-(2′,3′-dihydroxyphenyl)propanoic acid; 8, 3-(2′,4′-dihydroxyphenyl)propanoic acid; 9, 3-(3′,4′-dihydroxyphenyl)propanoic acid; 10, 3-(2′,5′-dihydroxyphenyl)propanoic acid; 11, 3-(3′,5′-dihydroxyphenyl)propanoic acid; 12a, b, 2*R/S*-hydroxy-3-(2′-hydroxyphenyl)propanoic acid; 13a, b, 2*R/S*-hydroxy-3-(3′-hydroxyphenyl)propanoic acid; 14a, b, 2*R/S*-hydroxy-3-(4′-hydroxyphenyl)propanoic acid; 15a–d, 2*R/S,3R/S*-dihydroxy-3-(phenyl)propanoic acid.

It is easy to overlook just how many isomers there might be even without the added complication of conjugation and geometric isomerization, because there may be regio- and stereo-isomers. Although perhaps not immediately obvious, there are ≥27 C_6_–C_3_ compounds having two hydroxyls and an exact mass of 182.0579 Da (**Figure 6**), and although they are not all typical urinary catabolites, it is important to be able to distinguish between them because each of these structures could have very different biological activities. Furthermore, two stereo-isomers are also possible for the C_6_–C_3_ phenylpropanoic acids, having a side-chain hydroxyl group at C-2 or C-3 (Figure 6, structures 1–6 and 12–15). Racemization—that is, conversion of an *S*-enantiomer to an *R*-enantiomer or the reverse—is possible at least for structures 1–3 in Figure 6 and is known to occur during mitochondrial β-oxidation of fatty acids, generally producing a small excess of the *R*-enantiomer (17). If it is uncertain which isomer is present, or if it is likely to be an unresolvable mixture, the structure can be described as, for example, 3*R*/*S*, and the structure can be drawn with the “wiggly” bond as shown in structures 1–6 and 12–15 (Figure 6). The *R* and *S* configuration can be critical in determining the interaction of a compound with a protein, transporter, or enzyme and hence is critical for biological activity. As an example of the importance of this structural discrimination, the *S*-enantiomer of ibuprofen is active as a painkiller but the *R*-enantiomer is completely inactive (18).

It is clear that the multiplicity of isomers is a problem, not only of nomenclature but also for the identification of metabolites in biological samples, because without the use of appropriate reference standards, the potential for misidentification is considerable. As noted previously, commercial vendors can often be lax with regard to nomenclature, and it is relatively common for samples to be impure or even incorrectly described. Even with appropriate reference standards, exact identification may not be possible if isomers are not chromatographically resolved. In such cases, extreme caution should be used when describing the metabolite, and using “tentative identification” or “partial identification,” rather than “identification,” is recommended. A feature of the older literature that is still valid today when the exact structure of a particular isomer is not known is to use the empirical formula (C_9_H_10_O_4_), a nominal molecular mass (182 Da), and a general name—for example, dihydroxy C_6_–C_3_ metabolite. In this example, all the compounds in Figure 5 would be encompassed because no positional assignment is provided for the hydroxyl groups. In such circumstances, the metabolic standards initiative proposals of Sumner et al. (16) are of value. From the previous account, it is clear that the multiplicity of synonyms and variations in nomenclature can be confusing for even the seasoned biochemist, and standardization is essential.

## Conclusion

The proposals made in this article will help establish a convenient, clear, and unambiguous nomenclature that is relevant to studies on microbiota-mediated breakdown of dietary (poly)phenols and their phase II metabolites. In publications, the use of fully characterized accurate names is recommended along with, whenever possible, ≥2 confirmatory identifiers taken from CAS numbers, InChIKey, and the IUPAC name, in addition to monoisotopic mass, because this allows others to locate the structure in question using recognized online databases. Furthermore, where analytical reference standards are not available and precise identification has not been possible, the metabolite should be described as fully as possible and the assignment downgraded to “tentative identification” or “partial identification.”

The proposed standardization of nomenclature will be of value to researchers because national funding bodies are beginning to require studies make their source data publicly available using open access data repositories. It will also be of value in literature searches for meta-analysis/systematic reviews. In such circumstances, standardized nomenclature will undoubtedly help researchers establish the untapped potential of (poly)phenols to human health.

## Supplementary Material

nqaa204_Supplemental_File

## Notes

CDK was supported by the USDA National Institute of Food and Agriculture (Hatch/Kay-Colin; 1011757). This work was partially supported by the JPI-HDHL FoodPhyt Project (CM by ANR-19-HDH2-0002-01 and PM and DDR by MIPAAF ID 1160) and partially by EU H2020 Project “PREVENTOMICS” (grant 818318) to PM and DDR. CA-L was supported by ISCIII (AC19/00096) and CIBERFES, co-funded by ERDF, “A way to make Europe,” ICREA Academia award 2018, and AGAUR (2017SGR1546).

Supplemental Table 1 is available from the “Supplementary data” link in the online posting of the article and from the same link in the online table of contents at https://academic.oup.com/ajcn/.
